# Utilizing small nutrient compounds as enhancers of exercise-induced mitochondrial biogenesis

**DOI:** 10.3389/fphys.2015.00296

**Published:** 2015-10-27

**Authors:** Daniel M. Craig, Stephen P. Ashcroft, Micah Y. Belew, Ben Stocks, Kevin Currell, Keith Baar, Andrew Philp

**Affiliations:** ^1^MRC Arthritis Research UK Centre for Musculoskeletal Ageing Research, School of Sport, Exercise and Rehabilitation Sciences, University of BirminghamBirmingham, UK; ^2^Molecular, Cell and Cancer Biology, University of Massachusetts Medical SchoolWorcester, MA, USA; ^3^EIS Performance Centre, English Institute of Sport, Loughborough UniversityLoughborough, UK; ^4^Neurobiology, Physiology and Behavior, University of California DavisDavis, CA, USA

**Keywords:** mitochondrial biogenesis, skeletal muscle, bioactives, nutraceuticals, exercise mimetics

## Abstract

Endurance exercise, when performed regularly as part of a training program, leads to increases in whole-body and skeletal muscle-specific oxidative capacity. At the cellular level, this adaptive response is manifested by an increased number of oxidative fibers (Type I and IIA myosin heavy chain), an increase in capillarity and an increase in mitochondrial biogenesis. The increase in mitochondrial biogenesis (increased volume and functional capacity) is fundamentally important as it leads to greater rates of oxidative phosphorylation and an improved capacity to utilize fatty acids during sub-maximal exercise. Given the importance of mitochondrial biogenesis for skeletal muscle performance, considerable attention has been given to understanding the molecular cues stimulated by endurance exercise that culminate in this adaptive response. In turn, this research has led to the identification of pharmaceutical compounds and small nutritional bioactive ingredients that appear able to amplify exercise-responsive signaling pathways in skeletal muscle. The aim of this review is to discuss these purported exercise mimetics and bioactive ingredients in the context of mitochondrial biogenesis in skeletal muscle. We will examine proposed modes of action, discuss evidence of application in skeletal muscle *in vivo* and finally comment on the feasibility of such approaches to support endurance-training applications in humans.

## Introduction

Endurance training leads to increased rates of fat oxidation and glycogen synthesis in skeletal muscle, which in turn contributes to enhanced endurance performance (Achten and Jeukendrup, 2004). As such, nutritional and training strategies aimed to maximize these adaptive responses have been an area of intense investigation. Even though traditional nutritional supplementation strategies were primarily aimed at prolonging endurance performance (Maughan, 2002), increased understanding of the molecular regulation of skeletal muscle adaptation during, and in response to exercise, have led to contemporary approaches utilizing pharmacological compounds, functional foods, or small naturally occurring bioactive ingredients to substitute for, or augment the training response. The aim of this review is to (1) discuss the benefits of regular exercise training on whole body and skeletal muscle-specific adaptation, (2) introduce the concept of exercise mimetics and discuss their feasibility in skeletal muscle *in vivo*, (3) critique the literature detailing the use of small nutritional bioactive ingredients as modulators of mitochondrial function in skeletal muscle *in vitro* and *in vivo*, and finally (4) discuss the efficacy of each approach for use in humans.

## Endurance exercise and aerobic adaptations

Regular physical activity in the form of endurance training can substantially improve endurance capacity in a range of populations (Ferketich et al., 1998; Gibala et al., 2006; Alemo Munters et al., 2013). This is achieved both by an increased maximal oxygen uptake ($\dot{\text{V}}$O_2_ max) and the ability to work at a given submaximal intensity with a smaller homeostatic disturbance (Bassett and Howley, 2000). Maximal oxygen uptake is principally governed by the delivery of oxygen to the musculature by the cardiovascular system and, to a lesser degree, the removal of oxygen from the blood at the exercising muscles (Saltin and Strange, 1992; Montero et al., 2015). Following regular bouts of aerobic exercise, left-ventricular hypertrophy, increased myocardial contractility, and increased end-diastolic volume increases stroke volume (Baggish et al., 2008; Bonne et al., 2014), with little to no difference in heart rate at maximal exercise intensities (Baggish et al., 2008; Murias et al., 2010; Bonne et al., 2014). Thus, maximal cardiac output increases as a result of greater stroke volume and is strongly related to the increase in $\dot{\text{V}}$O_2_ max (Jones and Carter, 2000).

At submaximal exercise intensities and at rest, the required cardiac output remains similar, however the elevated stroke volume results in a compensatory reduction in heart rate, known as bradycardia (Spina, 1999). Additionally, systemic resistance is decreased following aerobic exercise training (Klausen et al., 1982). This has the additional health benefit of decreasing blood pressure (Klausen et al., 1982), thereby reducing the risk of coronary heart disease and stroke (Morris et al., 1980; MacMahon et al., 1990; Lee et al., 2003).

Arterial oxygen carrying capacity is primarily determined by red blood cell and hemoglobin concentration. During aerobic training, initial decreases in hematocrit and hemoglobin concentration have been identified, which can be attributed to the rapid increase in plasma volume and does not reflect a decrease in red blood cell count (Sawka et al., 2000). In fact after several weeks of endurance training hematocrit returns to pre-training levels, despite greater plasma volume, indicating an increased hemoglobin and red blood cell volume and, therefore, oxygen carrying capacity of the blood (Sawka et al., 2000; Bonne et al., 2014). Aerobic training also results in an increased number and density of capillaries per muscle fiber (Ingjer, 1979; Murias et al., 2011), allowing for a more efficient and homogenous distribution of the increased cardiac output with little to no change in transit time through the musculature (Saltin, 1985; Kalliokoski et al., 2001). Increased capillary density results in shorter diffusion distances (Saltin and Rowell, 1980) and together with possible increases in myoglobin concentration within working muscles (Harms and Hickson, 1983) increases the oxygen extraction capacity of the musculature. This results in a greater arteriovenous oxygen difference across the working muscles (Beere et al., 1999; Murias et al., 2010) and therefore increases oxygen delivery for oxidative phosphorylation in skeletal muscle mitochondria at a given blood flow. Together, increased cardiac output and greater oxygen delivery and extraction in the exercising muscles increases $\dot{\text{V}}$O_2_ max (Spina, 1999).

While the cardiovascular system may limit maximal aerobic capacity, oxygen uptake at a given submaximal intensity is the same in the trained and untrained state (Hagberg et al., 1980). Exercise capacity at submaximal workloads is more closely related to adaptations in skeletal muscle (Bassett and Howley, 2000), which demonstrates considerable plasticity when exposed to different functional demands. Following endurance training, the shift in whole-body substrate oxidation toward greater lipid oxidation (Koivisto et al., 1982) and reduced glycolysis (Green et al., 1995) allows for a greater absolute exercise intensity to be supported predominantly by aerobic energy production. This results in reduced lactate accumulation in blood and muscle (Karlsson et al., 1972; Saltin et al., 1976; Bonen et al., 1998; Philp et al., 2008) and sparing of muscle glycogen stores at a given workload (Green et al., 1995), which play a pivotal role in the increased exercise capacity and performance following endurance training.

Endurance exercise training results in a shift toward a more oxidative, fatigue-resistant, phenotype of the trained muscle. An increased proportion of slow-twitch type I, fast oxidative type IIa, and hybrid fibers is apparent, with a reduction in rapidly fatiguing fast glycolytic type IIx and IIb fibers (Andersen and Henriksson, 1977; Simoneau et al., 1985; Fitts et al., 1989; Coggan et al., 1992). This is caused by hypertrophy of type I and type IIa fibers (Coggan et al., 1992) and a transformation of fibers to a slower phenotype, by an altered expression of myosin heavy chain isoforms (Putman et al., 2004). The shift toward a slower muscular phenotype is of physiological importance to endurance performance given the close relationship between muscle fiber composition and both the oxygen cost of locomotion and lactate threshold (Ivy et al., 1980).

Aerobic exercise promotes a large increase in mitochondrial mass, mitochondrial enzyme activity, and oxidation efficiency (Holloszy, 1967; Molé et al., 1971; Oscai and Holloszy, 1971; Hoppeler et al., 1973; Spina et al., 1996). Holloszy first demonstrated an increased mitochondrial enzyme activity in rats following progressive endurance training (Holloszy, 1967), a finding that has subsequently been replicated in numerous human studies (Gollnick et al., 1973; Hoppeler et al., 1973; Spina et al., 1996; Gibala et al., 2006; Little et al., 2010). The activity of enzymes in the electron transport chain, such as succinate dehydrogenase, NADH dehydrogenase, NADH-cytochrome-c reductase and cytochrome-c oxidase, can increase up to two-fold in response to training (Coggan et al., 1992). Concentrations of cytochrome-c also increase by approximately two-fold, suggesting the increased enzyme activity is due to an increase in mitochondrial enzyme protein content (Holloszy, 1967). Crucially, oxidative phosphorylation was tightly coupled, suggesting that the increase in electron transport capacity was associated with a proportional increase in the capacity for ATP production by oxidative phosphorylation (Holloszy, 1967). Enzymes involved in the citric acid cycle (Holloszy et al., 1970), fatty acid oxidation (Molé et al., 1971), and ketone oxidation (Winder et al., 1974) also increase. However, mitochondrial enzymes do not respond in a uniform manner to endurance training. In response to the same exercise stimulus in rats, enzymes involved in the oxidation of fatty acids increase by approximately two-fold (Molé et al., 1971), whereas enzymes of the citric acid cycle only increase by up to 50% (Holloszy et al., 1970). Glycolytic enzymes such as creatine phosphokinase, adenylate kinase, and α-glycerophosphate dehydrogenase remain unchanged, or even decrease in activity when expressed per milligram of mitochondrial protein content (Holloszy and Oscai, 1969; Oscai and Holloszy, 1971). Therefore, regular endurance exercise results in an adaptive response to increase the capacity for ATP resynthesis by oxidative phosphorylation, especially from the oxidation of fatty acids, and in doing so reduces the reliance upon glycolysis.

## Molecular regulation of skeletal muscle mitochondrial biogenesis

**Figure 1:**
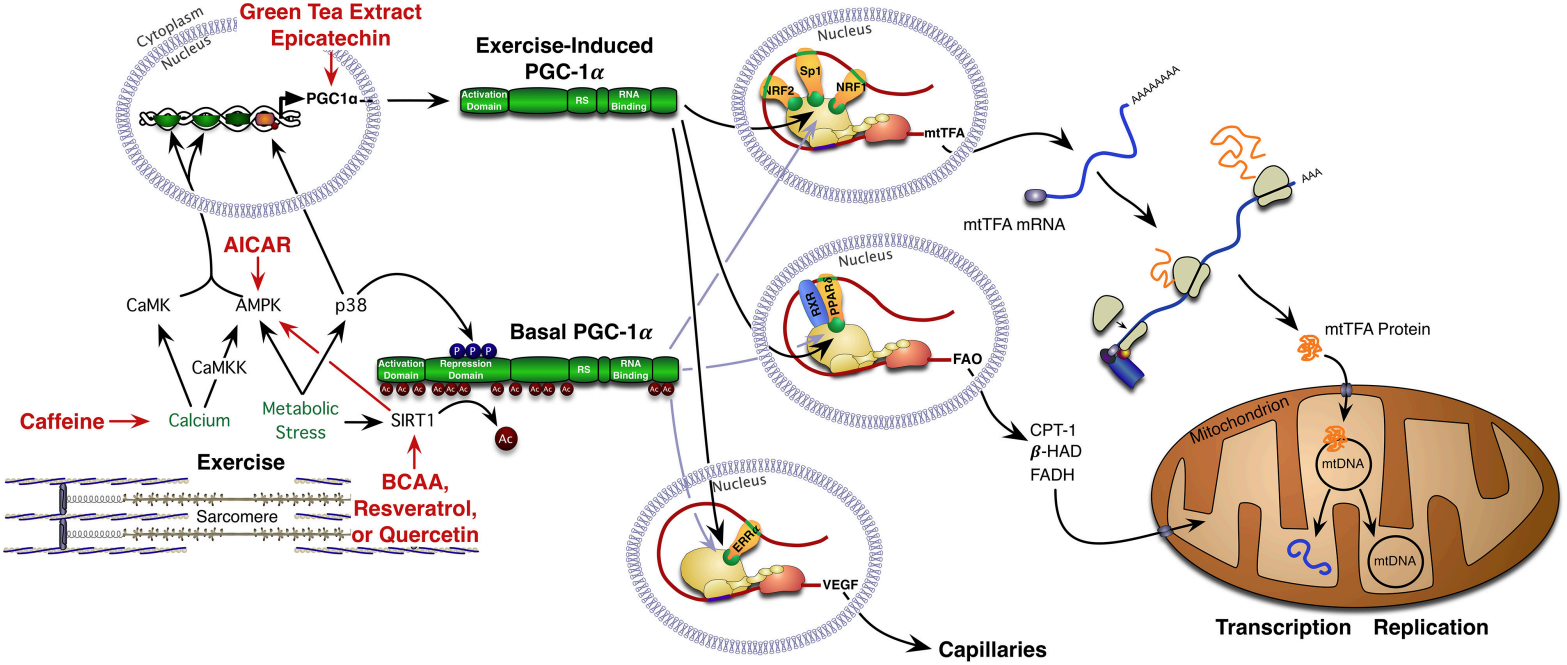
**Exercise-mediated mitochondrial biogenesis: exercise triggers mitochondrial biogenesis in skeletal muscle via the activation of numerous signaling pathways that ultimately converge on the transcriptional co-activator PGC-1α**. Once activated, PGC-1α translocates to the nucleus to activate numerous transcription factors and nuclear receptors. Bioactive compounds have the capacity to enhance exercise-mediated mitochondrial biogenesis through contraction-dependent signaling cascades. AICAR, 5-aminoimidazole-4-carboxamide ribonucleotide; AMPK, 5′ AMP-activated protein kinase; βHAD, beta-hydroxyacyl-CoA dehydrogenase; BCAA, branch chain amino acids; CaMK, Ca^2+^/calmodulin-dependent protein kinase; CaMKK, Ca(2+)/calmodulin-dependent protein kinase kinase; CPT-1, carnitine palmitoyltransferase I; ERRα, estrogen-related receptor alpha; FADH, flavin adenine dinucleotide; FAO, fatty acid oxidation; mRNA, messenger ribonucleic acid; mtDNA, mitochondrial deoxyribonucleic acid; mtTFA, mitochondrial transcription factor A; NRF1, nuclear respiratory factor-1; NRF2, nuclear respiratory factor-2; PGC-1α, peroxisome proliferator-activated receptor gamma coactivator 1-alpha; PPARδ, peroxisome proliferator-activated receptor delta; RS, RS domain; RXR, retinoid X receptor; SIRT1, sirtuin-1; Sp1, specificity protein 1 transcription factor; VEGF, vascular endothelial growth factor.

The driving stimulus by which exercise initiates mitochondrial biogenesis in skeletal muscle has been an area of intense investigation in the past decade. Current opinion is that alterations in the cellular environment, as a consequence of skeletal muscle contraction, are a principal signal driving the adaptive response (Figure 1). One of the most defined energy sensors in skeletal muscle is the adenosine monophosphate (AMP)-activated protein kinase (AMPK), an enzyme complex that is allosterically activated through increased AMP:adenosine triphosphate (ATP) ratios and phosphorylated via calcium dependent signaling pathways (Steinberg and Kemp, 2009). The activation of AMPK following exercise is intensity dependent with intensities of 60% VO_2_ peak reported to consistently induce activation (Chen et al., 2003). AMPK activity is amplified during exercise in a fasted or glycogen depleted state, following which it acutely stimulates increased rates of fat oxidation (Steinberg and Kemp, 2009). Once activated AMPK increases ATP production via an increase in lipid oxidation, by enhancing fatty acid uptake into skeletal muscle and increasing the transport of fatty acids into the mitochondria. The phosphorylation of acetyl-CoA carboxylase (ACC) by AMPK reduces the concentration of malonyl-CoA, which in turn reduces the allosteric inhibition of carnitine palmitoyltransferase 1 (CPT-1), allowing increased fatty acid transport into the mitochondria (Steinberg and Kemp, 2009).

In addition to the activation of AMPK, exercise also activates a number of other signaling intermediaries including, but not limited to, the nicotinamide adenine dinucleotide (NAD)^+^ dependent protein deacetylases Sirtuin 1 (SIRT1) and 3 (SIRT3) (White and Schenk, 2012), the tumor suppressor p53 (Bartlett et al., 2014), (p38 MAPK) (Lluis et al., 2006), and calcium calmodulin-dependent protein kinase II (CaMKII) (Wright, 2007). Briefly, exercise alters the cellular redox state in skeletal muscle leading to an increase in the NAD^+^:NADH ratio (White and Schenk, 2012). This subsequently results in an increase in the activation of SIRT1. SIRT1-mediated deacetylation of metabolic targets has been linked to transcriptional and post-translational regulation of intermediatory metabolism (White and Schenk, 2012). The tumor suppressor p53 has also been recently implicated in the regulation of mitochondrial function (Bartlett et al., 2014) given that following contraction, p53 has been reported to exhibit post-translational modification and alter its subcellular location (Saleem and Hood, 2013). Increases in the phosphorylation of p53, which is typically associated with an increase in activity and stability, have been reported in both rodent (Saleem and Hood, 2013) and human skeletal muscle (Bartlett et al., 2012) following exercise. The reported changes also act in a time course that could be related to the upstream signaling of either AMPK or p38MAPK (Bartlett et al., 2012). Following exercise, p38MAPK through a proposed calcium sensitive mechanism (Wright et al., 2007), is also activated and this regulates transcriptional events via phosphorylation (Bartlett et al., 2012). Numerous transcription factors and co-activators located in the cytosol and the nucleus are phosphorylated by p38MAPK, and loss of the gamma subunit of p38 blocks endurance training increases in mitochondrial biogenesis in mice (Pogozelski et al., 2009).

These signaling cascades ultimately converge on a host of co-activators and nuclear receptors that mediate the initiation of mitochondrial biogenesis (Perez-Schindler and Philp, 2015). Of note, the peroxisome proliferator-activated receptor-gamma co-activator (PGC-1α), a transcriptional co-activator that interacts with transcription factors at target gene promoters to increase transcriptional activity and promote mitochondrial remodeling (Puigserver et al., 1998) has received considerable attention in skeletal muscle. Transgenic activation of PCG-1α has been shown to mimic endurance training-induced adaptations including increased oxidative fiber content, mitochondrial biogenesis and angiogenesis (Yan et al., 2011), suggesting that activation of PGC-1α is a key driver of mitochondrial biogenesis is skeletal muscle. Further, following exercise the expression of PGC-1α shifts to an alternate promoter producing a smaller but more active form of the protein (Martínez-Redondo et al., 2015). The phosphorylation of PGC-1α by both AMPK and p38MAPK and deacetylation by SIRT1 are thought to increase its activity and translocation to the nucleus (Cantó and Auwerx, 2009; Dominy et al., 2010) (Figure 1). It is here that PGC-1α activates a number of transcription factors associated with mitochondrial biogenesis. These include nuclear respiratory factor 1 (NRF-1) and 2 (NRF-2), peroxisome proliferator-activated receptor (PPARδ), estrogen-related receptor (EERα), and myocyte enhancer factor 2 (MEF2) (Figure 1). In addition, PGC-1α translocation to the nucleus enhances the transcription of mitochondrial-encoded proteins via increased expression of mitochondrial transcription factor A (Yan et al., 2011) (Figure 1). It is the cumulative effect of these signaling cascades that result in long term adaptations in skeletal muscle including the increased abundance of proteins involved in mitochondrial ATP production, the tricarboxylic acid (TCA) cycle and the transport and oxidation of fatty acids.

## The scientific basis of exercise mimetics

Based on the premise that energy-sensing proteins such as AMPK and PPAR-δ are key signaling proteins modulating mitochondrial biogenesis in skeletal muscle, Narkar and coworkers examined the effect of administrating the PPAR-δ agonist GW501516 and the AMPK agonist 5-aminoimidazole-4-carboxamide ribonucleotide (AICAR) to mice (Narkar et al., 2008). The authors reported that GW501516 administration resulted in an increase in the mRNA expression of oxidative related genes such as Uncoupling Protein 3, CPT1, and Pyruvate Dehydrogenase Kinase 4, which was similar to the metabolic remodeling previously reported in PPAR-δ transgenic mice (Wang et al., 2004). However, despite the potent effects of GW501516 on oxidative gene expression, GW501516 treatment did not increase endurance capacity in supplemented mice (as was observed following exercise training), suggesting incomplete metabolic remodeling. Interestingly, GW501516 in combination with exercise training had synergistic effects on gene expression and endurance performance. In light of this observation, Narkar and colleagues substituted the AMPK agonist AICAR for exercise and reported a synergistic effect of the dual treatment strategy compared to either of the compounds in isolation. Unfortunately the authors did not compare this combined approach to endurance exercise, or the synergistic effect of the treatment with exercise. Based on this data, the authors proposed that AICAR and GW501516 are exercise mimetics and could be used as a strategy to increases skeletal muscle metabolism in the absence of an exercise stimulus. This concept received widespread attention and led to a boom in “exercise mimetic” therapy research (Matsakas and Narkar, 2010; Fan et al., 2013).

It should be noted that the use of AICAR to modulate skeletal muscle metabolism was not novel, in fact Will Winder's group had demonstrated the metabolic remodeling capacity of AICAR *in vivo* 11 years earlier (Merrill et al., 1997). It should also be highlighted that GW501516 was only effective in increasing endurance capacity when combined with exercise, and so cannot be regarded as an exercise mimetic, rather at best an exercise enhancer. To date, the work from Narkar and colleagues has failed to translate into human studies, mainly due the poor bioavailability of AICAR *in vivo* (Cuthbertson et al., 2007; Boon et al., 2008; Bosselaar et al., 2011). In addition, given that AICAR inhibits oxygen consumption in isolated muscle fibers (Spangenburg et al., 2013), the suitability of using of this compound *in vivo* is questionable. The efficacy of long-term GW501516 treatment has also been questioned due to links to cancer progression in number of tissues following chronic PPAR-δ activation (Sahebkar et al., 2014). The feasibility of an exercise mimetic has also raised considerable opposition in the literature (Goodyear, 2008; Richter et al., 2008; Carey and Kingwell, 2009), mainly due to the widespread, multi-organ health benefits of exercise (Hawley and Holloszy, 2009) that cannot be recapitulated with single-protein targeted therapeutics (Goodyear, 2008; Richter et al., 2008; Carey and Kingwell, 2009).

## Beyond exercise mimetics, can small bioactive ingredients enhance exercise-induced mitochondrial biogenesis?

Whilst the concept of an exercise mimetic, as proposed by Narkar and colleagues would appear to have a number of inherent flaws when it comes to *in vivo* application in humans (Goodyear, 2008; Richter et al., 2008), the use of functional foods or small bioactive ingredients to target exercise-responsive signaling networks does appear to hold promise (Crowe et al., 2013). Typically, bioactive ingredients are viewed as both essential and non-essential compounds (e.g., vitamins or polyphenols) that occur in nature and can be shown to have an effect on human health. Whilst bioactive ingredients are already known to have far-reaching health benefits (Crowe et al., 2015), there is limited information with specific regard to skeletal muscle mitochondrial biogenesis. In the following sections we will briefly highlight a selection of bioactives and when appropriate discuss their proposed mode of action and efficacy/feasibility for translating this research into human-based investigation.

### Green tea extracts (GTEs)

GTEs are a class of polyphenolic flavonoids which are suggested to play a role in fatty acids (FA) mobilization and oxidation (Shimotoyodome et al., 2005). The polyphenolic compounds in GTEs are epigallocatechin gallate (EGCG), epicatechin gallate (ECG), and gallocatechin gallate (GCG). EGCG is suggested to be the most pharmacologically active; between 210 and 760 times potent as the others (Zhu et al., 2008). GTEs have been suggested to modulate fat oxidation via altered catecholamine release, with Dulloo et al. (1999) demonstrating greater 24-h basal energy expenditure (EE) following GTE supplementation compared to caffeine or a placebo. In addition, they observed a higher percentage of fat-derived 24-h EE compared to the other groups (Dulloo et al., 1999). In support, Venables et al. (2008) demonstrated an increased FA oxidation rate in GTE treated participants vs. a placebo group during exercise, indicated by increased circulating free fatty acids (FFAs) and glycerol (Venables et al., 2008). In this study plasma glucose and insulin concentrations were concurrently lower in the GTE group, indicating a significant metabolic shift toward lipid oxidation (Venables et al., 2008). More recently, Hodgson et al. (2013) and Randell et al. (2013) demonstrated that 7 days GTE supplementation altered global metabolite profiles and increased lipolysis (Randell et al., 2013).

In contrast, (Randell et al., 2013) recently failed to fully reproduce the data from Venables et al. (2008), demonstrating no effect of GTE supplementation on fat oxidation during exercise. A follow up study by the same group (Randell et al., 2014) also demonstrated that de-caffeinated GTE supplementation over 1, 7, and 28 days had no effect on whole-body fat oxidation or fat metabolism-related metabolites during exercise (Randell et al., 2014). Thus, it is currently unclear whether GTE supplementation alone is enough to alter fat oxidation. A small number of studies have been performed to investigate the chronic effects of GTE supplementation on fat oxidation. Based on acute study data, it could be suggested that longer-term GTE supplementation may result in greater increases in fat oxidation. In mice, GTE ingestion during 15 weeks of regular exercise significantly lowered respiratory exchange ratio (RER) and also increased fat utilization compared to an exercise-only group (Shimotoyodome et al., 2005). In humans, Ota et al. (2005) demonstrated that combined GTE supplementation and endurance exercise elicited 24% higher FA oxidation compared to a placebo supplement (Ota et al., 2005). Combined with findings of acute studies, these data lend support to the argument that GTE supplementation with exercise may be efficacious in improving fat oxidation. However, an optimal supplementation strategy is yet to be defined.

Regarding mechanisms of action, Murase et al. (2006) observed that GTE supplementation led to increased activation of PGC-1α mRNA in skeletal muscle and parallel increases in treadmill running time in mice (Murase et al., 2006). As such, it is hypothesized that GTEs may increase mitochondrial biogenesis and skeletal muscle FA oxidation through a PGC-1α-dependent pathway however this has yet to be directly tested.

### Caffeine

Caffeine has been shown to stimulate sympathetic nervous system (SNS) activity (Graham et al., 2000) and increase norepinephrine (NE) at the synaptic junction (Dulloo et al., 1992). Caffeine also harnesses the potential to inhibit phosphodiesterase, a cyclic AMP (cAMP) degrading enzyme (Dulloo et al., 1999). Thus, intracellular cAMP may increase with caffeine ingestion promoting subsequent rises in catecholamine concentrations (Dulloo et al., 1999). In addition, caffeine has been reported to stimulate intracellular calcium release (Youn et al., 1991) to a similar extent to contracted skeletal muscle (Baar, 2006). This increased calcium flux activates an upstream kinase to AMPK, calmodulin kinase kinase (Egawa et al., 2011). The caffeine-induced activation of AMPK has also been shown to increase insulin-dependent uptake of glucose similarly to exercise-induced activation (Egawa et al., 2009). It is therefore suggested that caffeine may promote fat oxidation through increased thermogenesis (via AMPK) possibly via increased SNS activity.

Graham et al. (2000) demonstrated that following steady-state exercise at 70% $\dot{\text{V}}$O_2_ max and caffeine supplementation, serum FA and glycerol concentration was increased compared to a placebo, but that no differences in RER or FA uptake were found (Graham et al., 2000). Acheson et al. (2004) showed that caffeine supplementation increased FA disposal and EE during steady-state cycling exercise. They demonstrated that lipid turnover was markedly increased, but only small increments in FA oxidation were observed (Acheson et al., 2004). In both cases, the results suggest that caffeine supplementation stimulated the SNS, but had minimal “downstream” effects on FA oxidation; despite increased FA utilization. To our knowledge, there have been no studies directly examining the intra-muscular signaling related to caffeine supplementation, or whether caffeine can alter mitochondrial biogenesis in skeletal muscle.

### Epicatechins

Cocoa-derived epicatechins, specifically (−)-epicatechin, have been shown to activate mitochondrial biogenesis and capillary proliferation in murine skeletal muscle, in addition to having multiple health benefits in humans (Buijsse et al., 2010). Nogueira et al. (2011) were the first to demonstrate that 15 days (−)-epicatechin supplementation in mice increased treadmill running performance, fatigue resistance, mitochondrial volume, and muscle capillarity in mice compared to activity-matched control groups (Nogueira et al., 2011). These results suggest that (−)-epicatechin supplementation independently resulted in a response similar to endurance exercise as well as augmented the endurance exercise training response, therefore epicatechin has the potential to promote metabolic changes within skeletal muscle resulting in mitochondrial biogenesis. A second study by the same group (Huttemann et al., 2012) investigated the influence of (−)-epicatechin supplementation in mice undergoing 5 weeks of endurance training and then 2 weeks of de-training. Of interest, the epicatechin treated group maintained capillary-fiber ratio and cytochrome-c oxidase activity after the de-training period suggesting that (−)-epicatechin may maintain the endurance-training effect through specific up regulation of angiogenesis and mitochondrial biogenesis pathways (Huttemann et al., 2012).

The first translation of these rodent studies into human investigation was recently performed by Gutiérrez-Salmeán et al. (2014), who investigated the effects of epicatechin supplementation on post-prandial fat metabolism in normal and overweight adults (Gutiérrez-Salmeán et al., 2014). Following supplementation of (−)-epicatechin (1 mg/kg), participants displayed a lower RER, indicative of increased lipid oxidation. In addition, lower plasma glucose concentrations were observed following the supplementation (Gutiérrez-Salmeán et al., 2014). From the available data, it is suggested the cocoa-derived epicatechin is a promising ergogenic aid for increasing mitochondrial biogenesis and lipid oxidation, with the nitric-oxide (NO)/vascular endothelial growth factor (VEGF) pathway suggested to be the primary molecular mechanisms linking (−)-epicatechin supplementation to enhanced muscle adaptation. It will be interesting to see whether the work from Nogueira et al. (2011) can be recapitulated in humans and enhance mitochondrial adaptation to endurance exercise training.

### Polyphenols

Polyphenol compounds are found in a variety of herbal medicines commonly used as ethnopharmaceutical agents. These compounds have attracted the attention of researchers owing to their cardio-protective qualities (Chong et al., 2010). Two compounds in particular (resveratrol and quercetin) have been recently studied, and their relevance to fat oxidation during endurance exercise and mitochondrial adaptation to training is discussed here:

#### Resveratrol

Resveratrol is a stilbenoid polyphenol, a molecule belonging to the phenylpropanoid family commonly found in red wine. During the past decade, resveratrol has emerged as a potent cardio-protective compound. It is also associated with reduction in ischemic injuries and incidence of cancer. In 1992, resveratrol was first isolated from red wine (Siemann and Creasy, 1992) and subsequently attracted significant attention from researchers. It has since been demonstrated that it targets various signaling molecules that work to promote fat metabolism (Lopez-Lluch et al., 2008), and thus could be considered as a potential ergogenic aid.

Howitz et al. (2003) suggested that resveratrol may mimic the effect of caloric restriction by stimulating SIRT1 (Howitz et al., 2003). The mechanism by which resveratrol is able to increase fat oxidation is considered to be via SIRT1-dependent activation of AMPK (Lin et al., 2010), although SIRT1/AMPK independent pathways of resveratrol action have also been reported (Park et al., 2012). Smith et al. (2009) demonstrated that formulated resveratrol (SIRT501 and SRT1720) induced a signaling profile mirroring a reduction in type 2 diabetes pathology, which includes mitochondrial biogenesis and an improved metabolic signaling pathway (Smith et al., 2009).

Resveratrol has been shown to promote fat oxidation and enhance endurance performance in mice (Murase et al., 2009). However, Scribbans et al. (2014) recently reported that resveratrol supplementation during exercise training in healthy individuals led to a maladaptive response in exercise-stimulated gene expression (Scribbans et al., 2014). In agreement with this observation, Gliemann et al. (2013) showed that resveratrol supplementation in combination with high-intensity training in older men not only blunted the increase in maximal oxygen uptake observed in the placebo group, but also eradicated the effects of the exercise on low-density lipoprotein, total cholesterol, and triglyceride concentrations in the blood (Gliemann et al., 2013). Using a similar protocol, Olesen et al. (2014) recently showed that resveratrol supplementation also blunted training-induced decreases in protein carbonylation and tumor necrosis factor α (TNFα) mRNA within older individuals' skeletal muscle (Olesen et al., 2014). Thus, there are clear discrepancies between cell, rodent, and human studies investigating resveratrol supplementation. Given these discrepancies further studies are clearly warranted to understand the interaction between resveratrol and exercise in skeletal muscle, and begin to develop optimal supplementation strategies for improving mitochondrial biogenesis and fat oxidation during endurance exercise.

#### Quercetin

Quercetin is the most ubiquitous class of flavonoids; found in apples, onions, berries, leafy green vegetables, hot peppers, red grapes, and black tea. It has a similar structure and function to resveratrol, and numerous positive effects on skeletal muscle have been reported (Davis et al., 2009a). Though quercetin has a similar structure and function to resveratrol, it is classified as a different polyphenol owing to its ketone-containing structure (Bravo, 1998).

Quercetin supplementation has been reported to increase mitochondrial biogenesis and exercise tolerance in mice, through a proposed SIRT1/AMPK/PGC-1α mode of action (Davis et al., 2009b). Quercetin is suggested to act as an ergogenic aid by mimicking the effects of caffeine and its ability to activate Adenosine (A1) receptor (Alexander, 2006), by increasing AMPK activation (Hawley et al., 2010) and also by increasing SIRT1 gene expression and activation (Howitz et al., 2003). By decreasing ATP concentration, quercetin may activate AMPK by increasing the AMP:ATP ratio. This has been observed in isolated mitochondria (Dorta et al., 2005), and is thought to induce this in a manner similar to resveratrol (Hawley et al., 2010).

Quercetin increases β-oxidation in hepatocytes (Suchankova et al., 2009), C2C12 muscle cells (Eid et al., 2010), and Hela cells (Jung et al., 2010). However, short-term quercetin supplementation (1000 mg/d) in humans does not affect $\dot{\text{V}}$O_2_ max (Ganio et al., 2010), whereas prolonged oral supplementation (3 weeks of 1000 mg/d) elicited no change in RER or fat oxidation (Dumke et al., 2009). Similarly, Cureton et al. (2009) observed no difference in exercise performance or substrate shifts in recreationally active participants (Cureton et al., 2009). In contrast, Nieman et al. (2010) reported small improvements in endurance performance and PGC-1α, SIRT1, citrate synthase and cytochrome-c oxidase mRNA in untrained participants supplemented with quercetin (Nieman et al., 2010).

From the available evidence, it is suggested that quercetin supplementation has little or no effect on mitochondrial biogenesis/fat oxidation in human skeletal muscle performing endurance exercise. However, similar to resveratrol, there are only a paucity of studies that have investigated this and the apparent discrepancies between the rodent and human studies have yet to be fully elucidated.

### Amino acids

A substantial body of evidence now supports the use of protein and amino acids for skeletal muscle training adaptation to resistance training programs aimed at increasing muscle mass (Atherton and Smith, 2012). However, in comparison, the role of amino acids in mitochondrial biogenesis/fat oxidation is under-represented in the literature.

Branch chain amino acid (BCAA) supplementation (particularly leucine) has become popular amongst athletes and recreational exercisers owing to its established role in promoting muscle protein synthesis and positive changes in body composition. A high-protein diet elicits greater levels of resting fat oxidation compared with an iso-energetic high-carbohydrate or high-fat meal (Raben et al., 2003). In addition, Labayen et al. (2004) showed that a single high-protein meal could induce an increase in post-prandial fat over 6 h compared to a standard mixed meal in both lean and obese women. Furthermore, post-prandial fat oxidation, protein and leucine oxidation were greater following the high-protein meal (Labayen et al., 2004). This increased fat oxidation rate may be explained by BCAA (specifically, leucine) oxidation.

BCAA transaminase activation has been shown to occur concurrently with exercise-induced glycogen depletion (Gualano et al., 2011), suggesting a possible role for BCAA in lipid oxidation. Accordingly, a study performed by Gualano et al. (2011) investigated the influence of BCAAs on fat oxidation and exercise capacity during endurance exercise. They demonstrated that BCAA supplementation induced a lower RER during the exhaustive exercise test, and promoted a greater resistance to fatigue (Gualano et al., 2011). These results lend further support to the argument that BCAA supplementation may promote increased fat oxidation during exercise.

Regarding BCAA action at the cellular level, Sun and Zemel (2009) reported that leucine administration to C2C12 cells exhibited increased mitochondrial mass, stimulated PGC-1α and SIRT1 gene expression and increased cell respiration (Sun and Zemel, 2009). These data suggest that leucine modulation of muscle energy metabolism may be mediated by mitochondrial biogenesis. Similarly, muscle cells treated with serum from overweight subjects fed a high-dairy diet for 28 days resulted in increased SIRT1 and PGC-1α expression *in vitro* (Bruckbauer and Zemel, 2011). These data suggest that high dairy consumption (thus, high leucine and BCAA consumption) may promote mitochondrial biogenesis within skeletal muscle. In mice, it has been shown that BCAA ingestion increased mitochondrial biogenesis and SIRT1 expression in skeletal muscle, which consequently increased lifespan in middle-aged mice (D'Antona et al., 2010). It was since hypothesized that leucine-induced activation of SIRT1 was a central event that linked the mitochondrial biogenesis and fat oxidation within skeletal muscle (Liang et al., 2014). To test this, Liang et al. (2014) treated C2C12 myotubes with leucine, and observed significantly increased mitochondrial content, fat oxidation and SIRT1 activity following this treatment compared to control treatments. In addition, time-dependent increases in NAD^+^ and SIRT1 activity were observed after 24-h leucine treatment (Liang et al., 2014). Beyond *in vitro* approaches, there are few studies examining the direct effect of amino acids on mitochondrial biogenesis in skeletal muscle. Given the efficacy of AAs for human supplementation (Moore et al., 2014), and the known benefits of AAs in recovery from endurance exercise (Moore et al., 2014), extending this research into the regulation of mitochondrial biogenesis seems the next logical progression in this research area (Moore and Stellingwerff, 2012).

## Conclusions

Endurance exercise is a potent stimulus to induce mitochondrial biogenesis in skeletal muscle (Holloszy, 1967; Molé et al., 1971; Oscai and Holloszy, 1971; Hoppeler et al., 1973; Spina et al., 1996). The nutritional approaches described herein could provide a framework to support endurance training via enhancing mitochondrial biogenesis. In this context, we propose that these small molecules should be viewed as exercise enhancers, not mimetics, as they have minimal effect in basal conditions. In the future, it will be interesting to explore the efficacy of using these nutrients in human studies *in vivo*, to identify the exercise setting in which they may have the most benefit as well as developing optimal supplementation strategies. In this regard, future studies could examine the effect of bioactives during and in recovery from exercise across a variety of intensities, and also examine supplementation during periods of tapering or detraining to shed light on the practical implications of bioactives as regulators of mitochondrial biogenesis in skeletal muscle. In order to achieve this, researchers should perform randomized, placebo-controlled, intervention trials in human subjects (Hasler, 2002), and examine the extent to which the bioactive ingredient in question is absorbed and bioavailable in skeletal muscle (Crowe et al., 2013). Once achieved, it is hoped that bioactives such as those discussed, and derivatives/associated bioactive ingredients yet to be identified may lead to the next-generation of nutritional supplements to specifically enhance mitochondrial adaptations to endurance training.

### Conflict of interest statement

Keith Baar has received research support from Sirtris pharmaceuticals and PepsiCo. Andrew Philp has received research support from Lucozade Ribena Suntory and Rank prize funds nutrition. Keith Baar and Andrew Philp are shareholders of Advanced Muscle Technologies. The authors declare that the research was conducted in the absence of any commercial or financial relationships that could be construed as a potential conflict of interest.
